# Regeneration of critical-sized defects, in a goat model, using a dextrin-based hydrogel associated with granular synthetic bone substitute

**DOI:** 10.1093/rb/rbaa036

**Published:** 2020-11-28

**Authors:** Isabel Pereira, José Eduardo Pereira, Luís Maltez, Alexandra Rodrigues, Catarina Rodrigues, Manuela Oliveira, Dina M Silva, Ana Rita Caseiro, Justina Prada, Ana Colette Maurício, José Domingos Santos, Miguel Gama

**Affiliations:** 1 CEB, Centre of Biological Engineering, University of Minho, Campus de Gualtar, Braga 4710-057, Portugal; 2 CECAV, Animal and Veterinary Research Centre, University of Trás-os-Montes e Alto Douro, Vila Real 5001-801, Portugal; 3 Department of Veterinary Sciences, University of Trás-os-Montes e Alto Douro, Vila Real 5001-801, Portugal; 4 Biosckin, Molecular and Cell Therapies S.A., Laboratório Criovida, TecMaia, Rua Engenheiro Frederico Ulrich 2650, Moreira da Maia 4470-605, Portugal; 5 Departamento de Clínicas Veterinárias, Instituto de Ciências Biomédicas de Abel Salazar (ICBAS), Universidade do Porto (UP), Rua de Jorge Viterbo Ferreira, n° 228, Porto 4050-313, Portugal; 6 Centro de Estudos de Ciência Animal (CECA), Instituto de Ciências, Tecnologias e Agroambiente da Universidade do Porto (ICETA), Rua D. Manuel II, Apartado 55142, Porto 4051-401 Portugal; 7 Centro de Investigação Vasco da Gama (CIVG)/Escola Universitária Vasco da Gama (EUVG), Avenida José R. Sousa Fernandes, n.° 197 Lordemão, Coimbra 3020-210, Portugal; 8 REQUIMTE/LAQV, Departamento de Engenharia Metalúrgica e Materiais, Faculdade de Engenharia, Universidade do Porto, Rua Dr Roberto Frias, Porto 4200-495, Portugal

**Keywords:** polysaccharide, injectable hydrogel, granular ceramics, Bonelike^®^, bone regeneration, calvarial defect

## Abstract

The development of injectable bone substitutes (IBS) have obtained great importance in the bone regeneration field, as a strategy to reach hardly accessible defects using minimally invasive techniques and able to fit to irregular topographies. In this scenario, the association of injectable hydrogels and bone graft granules is emerging as a well-established trend. Particularly, *in situ* forming hydrogels have arisen as a new IBS generation. An *in situ* forming and injectable dextrin-based hydrogel (HG) was developed, aiming to act as a carrier of granular bone substitutes and bioactive agents. In this work, the HG was associated to a granular bone substitute (Bonelike^®^) and implanted in goat critical-sized calvarial defects (14 mm) for 3, 6 and 12 weeks. The results showed that HG improved the handling properties of the Bonelike^®^ granules and did not affect its osteoconductive features, neither impairing the bone regeneration process. Human multipotent mesenchymal stromal cells from the umbilical cord, extracellular matrix hydrolysates and the pro-angiogenic peptide LLKKK18 were also combined with the IBS. These bioactive agents did not enhance the new bone formation significantly under the conditions tested, according to micro-computed tomography and histological analysis.

## Introduction

Increasing life expectancy and population ageing has raised the incidence of bone fractures, as well as the interest in bone regeneration research [1]. Conventional treatments (auto-, allo- and xenograft) present drawbacks such as limited supply, adverse immunological responses, disease transmission and morbidity [1, 2]. The lack of a simultaneously effective and safe treatment is pushing the development of synthetic bone substitutes (SBSs) [1, 3]. Glass-reinforced hydroxyapatite (HAP) composites are calcium phosphate-based substitutes able to chemically mimic the bone inorganic structure composition. Its osteoconductive property creates an ideal environment for cell attachment and proliferation, thus promoting bone regeneration [4–6]. However, many of commercially accessible SBSs are available in granular form [1] are difficult to handle and to fit in irregularly shaped defects. Also, granules can be washed out from the bone defect and, consequently, migration into the surrounding tissues with adverse or unexpected events [3, 7, 8]. Moreover, the micromovements of the granules within the defect can affect the formation of new bone tissue.

Attempting to solve these issues and improve their applicability in the surgical theatre, granular bone grafts have been combined with hydrogels to achieve injectable/mouldable SBSs, able to ensure granules cohesiveness, site retention and adaptability to the defect area [9–11]. In addition, injectable bone substitutes (IBSs) may be advantageous in some clinical applications where access to the bone defects is restricted, such as the spine injuries and maxillofacial defects [12]. The hydrogel can also turn the SBS osteoinductive and osteogenic through the incorporation of bioactive molecules or cellular systems [9, 10]. In particular, *in situ* gelling hydrogels have the advantage to improve the bioactivity of the IBSs, since they enable the homogeneous dispersion of the cellular systems and bioactive molecules, immediately before the crosslinking reaction. These systems also allows the *in situ* tailoring of the formulation, by the incorporation of specific therapeutics according to the needs of each clinical case [10]. In this context, it has been developing and characterizing by our research group an injectable dextrin-based hydrogel (HG), which can be loaded with stem cells, micro/nanoparticles and biomolecules, during clinical procedures [13–17]. In previous studies, the HG demonstrated *in vitro* cyto- and genocompatibility [14, 16]. Moreover, in *in vivo studies*, carried out in rodent models, the results showed that HG is biodegradable, does not induce any local and systemic toxic effect or skin reaction, and displays bone histocompatibility [15, 17].

The extracellular matrix (ECM) comprehends a collection of functional and structural biomolecules secreted by the resident cells that are responsible for the intrinsic mechanical and biochemical cues which regulate cell behaviour and function, as well as homeostasis and response to injury [18]. Thus, ECM has been applied as biological scaffolds for tissue engineering applications [19]. *In vitro* studies have demonstrated that the degradation products from ECM (obtained through chemical, enzymatic and heat treatment) display chemotactic and mitogenic activity on stem and progenitor cells [20–26], as well as antimicrobial activity [27, 28]. We hypothesize that the incorporation of ECM degradation products in the HG could improve the bioactivity of the IBS systems.

Vascularization is a pivotal process in the bone regeneration process and a limiting step in the healing of large bone defects [29, 30]. In this scenario, we hypothesized that the inclusion of angiogenic factors within the IBS systems can facilitate and enhance bone regeneration. LL37 is an antimicrobial peptide with 37 amino acid residues belonging to the family of cathelicidins [31]. Beyond its antimicrobial activity, some studies have been demonstrated that LL37 is involved in mast cells and leukocytes chemotaxis, angiogenesis and in wound healing promotion [31–33]. Recent studies have reported that LL37 can accelerate the bone regeneration process by promoting angiogenesis and subsequent recruitment of multipotent mesenchymal stromal cells (MSCs) into the bone defect [34], as well as inhibition of osteoclastogenesis [35]. LL37 derivatives have been engineered to increase its antimicrobial property and to decrease its cytotoxicity for human cells and binding to plasma protein [36, 37]. One of them, LLKKK18 presents higher chemoattractant activity [36] and preserved the ability to improve skin wound healing [38]. However, to date no significant data are available regarding LLKKK18 effect on bone regeneration.

MSCs are an important tool in regenerative medicine due to their intrinsic characteristics, such as, chemotactic, multilineage differentiation and immunogenic and inflammatory modulation capacities [39]. Human MSCs (hMSCs) have been defined by the International Society for Cellular Therapy ISCT [40] as being plastic adherent in standard culture conditions, which express specific cluster of differentiation (CD) 105^+^, 73^+^, 90^+^ and lack expression of haematopoietic markers: CD45^+^, CD34, CD14 or CD11b, CD79α or CD19 and HLA-DR surface molecules. The absence of expression of HLA-DR surface molecules enables the xenogeneic application of hMSCs, without the risks of immunogenic reactions. MSCs derived from the umbilical cord tissue represent a potent MSC source due to their youth, as they derive from a tissue collected at birth, in an easy and non-invasive procedure. Also, the quality and availability of cryopreserved tissue in private and public cryopreservation banks represents an advantage regarding the application of this cell source. Several reports have shown the beneficial properties of MSCs in bone tissue regeneration, not only through their differentiation potential but more importantly due to paracrine secretion of cytokines and growth factors modulating tissue repair process [41–44].

The main aims of this work were (i) to assess the association of the dextrin-based HG with granular ceramics (250–500 µm) on the regeneration of calvarial critical-sized defects, using a goat model and (ii) to evaluate the effect of porcine small intestinal submucosa (SIS) enzymatic degradation products, LLKKK18 and umbilical cord-derived hMSCs on bone regeneration, when combined with the dextrin-based IBS system.

## Material and methods

### Materials and reagents

Dextrin used in this study was Tackidex B 167 (Batch E 1445), a generous gift from Roquette (Lestrem, France). ECM from porcine SIS was a gift from Cook Biotech Inc. (West Lafayette, IN, USA). LLKKK18 (KEFKRIVKRIKKFLRKLV) was purchased from Schafer-N (Copenhagen, Denmark). Sodium *m*-periodate, diethylene glycol, adipic acid dihydrazide (ADH), silver nitrate, sodium thiosulphate, dexamethasone, ascorbic acid-2-phosphate, β-glycerphoshate, Alcian Blue, acetic acid, sodium carbonate (Na_2_CO_3_), calcium fluoride (CaF_2_), calcium hydrogen phosphate (CaHPO_4_), di-phosphorus penta-oxide (P_2_O_5_) and polyvinyl alcohol, formaldehyde, pepsin and hydrochloric acid (HCl) were purchased from Sigma-Aldrich (St. Louis, MO, USA). Dulbecco's Modified Eagle Medium (DMEM) with GlutaMAX™ and without nucleosides, foetal bovine serum, streptomycin, penicillin, amphotericin B, HEPES Buffer solution, StemPro^®^ Adipogenesis Differentiation kit and StemPro^®^ Chondrogenesis Differentiation kit were obtained from Gibco^®^. Accutase^TM^ Cell detachment solution, Stain Buffer and CD90, CD105, CD44, CD34, CD19, CD11b, CD45, MHC Class II antibodies were obtained from BD Biosciences©.

### Dextrin oxidation

Dextrin was oxidized according to the method previously described by our group [45]. Briefly, dextrin (2% w/v) was oxidized in the dark with sodium *m*-periodate, to yield the theoretical degree of oxidation of 40%, with stirring, for 20 h, at room temperature. The oxidation reaction was stopped with diethylene glycol. Sodium *m*-periodate and diethylene glycol were taken out by ultrafiltration (cut-off 1000 Da), and lyophilized.

### Preparation of dextrin-based hydrogel

HG was prepared as described by Pereira *et al*. [16]. Oxidized dextrin (ODEX) solutions were prepared in phosphate-buffered saline (PBS) buffer (30% w/v) and sterilized by gamma irradiation (20 kGy; 2 kGy/h), by IONISOS (Dagneux, France). ADH solutions were prepared in PBS buffer (3.76% w/v) and sterilized by filtration. For crosslinking reaction, ADH and ODEX solutions were mixed in a volume ratio of 3:7, before implantation in calvarial critical-sized defects.

### Preparation of bonelike^®^ granules

For the preparation of Bonelike^®^ (BL), HAP powder was mixed with a low temperature P_2_O_5_-CaO-based glass with the chemical composition of 65P_2_O_5_-15CaO-10CaF_2_-10Na_2_O (mol%) [46, 47]. For the glass preparation, appropriate quantities of Na_2_CO_3_, CaHPO_4_, CaF_2_ and P_2_O_5_ reagents were placed in a platinum crucible, heated up to 1450°C, for 90 min in a glass furnace and poured to deionized water. The poured glass was finally crushed in an agate mortar and sieved to obtain a fine glass powder with a particle size below 50 μm. This glass was mixed with the HAP powder, in a weight proportion of 97.5% of HAP and 2.5% of the glass. These two powders were then mixed with microcrystalline cellulose and polyvinyl alcohol, which both act as pore formers to obtain the macroporous structure. The resulting suspension was poured into Alumina (Al_2_O_3_) plates, dried at 60°C for 2 days and samples were sintered at 1300°C using a heating rate of 4°C/min, a dwell time of 1 h followed by natural cooling. Finally, BL granules with particle size between 250 and 500 μm were obtained, displaying a macroporous structure with interconnective porosity.

### Enzymatic hydrolysis of SIS matrix

This procedure was carried out as reported before [14]. Briefly, 150 mg of powdered SIS was digested with 15 mg of pepsin in 15 ml of 0.01 M HCl, during 48 h, under stirring, at 37°C. After the pepsin inactivation, the hydrolysates were lyophilized.

### Human multipotent MSCs culture and characterization

hMSCs from the umbilical cord stroma were purchased to PromoCell. hMSCs were thawed and expanded *in vitro*, using as culture medium, DMEM, with GlutaMAX™, without nucleosides supplemented with 10% (v/v) foetal bovine serum, 10 mM HEPES Buffer solution, 0.1 mg/ml streptomycin, 100 IU/ml penicillin and 2.05 µg/ml amphotericin B. The confirmation that the obtained cells were MSCs, was performed by flow cytometry and trilineage differentiation capacity. Flow cytometry was performed as described previously [39]. Briefly, passage 5 cultures (P5) were incubated with anti-positive (CD90, CD44, CD105) and anti-negative marker (CD11b, CD34, CD19, CD45, MHC Class II) antibodies (Human MSC Analysis Kit). Analysis was performed using a BD FACSCalibur™ 3 CA Becton Dickinson, and acquired data were processed using FlowJo Engine X10.4 (v3.05478, LLC). For trilineage differentiation capacity, hMSCs were seeded onto 24-well plates (8000 viable cells/cm^2^) and then transitioned onto specific differentiation media for adipogenesis (StemPro^®^ Adipogenesis Differentiation Kit) and osteogenesis (standard culture medium supplemented with 250 µM ascorbic acid-2-phosphate, 5 nM Dexamethasone and 10 mM β-glycerphoshate) and the undifferentiated controls were maintained in standard media. Every 3 days, differentiation and standard media was refreshed. After 14 days, adipogenic differentiation cells were stained with oil red O, for lipid droplet detection. Von Kossa staining was performed to observe mineral extracellular deposition. Cells were fixed and dehydrated with increasing ethanol concentrations. Then, cells were rehydrated and incubated with 2% silver nitrate solution, under ultra-violet light and sodium thiosulphate 5% for 3 min. Wells were rinsed, and photographic record obtained. For chondrogenic differentiation, hMSCs were seeded onto a 96-well plate (2 × 10^4^ viable cells/well) for 48 h, and then transferred to chondrogenic culture medium (StemPro^®^ Chondrogenesis Differentiation Kit) while control wells remained in standard culture medium. Media were renewed every 2 days, for 14 days. After that time, cells were fixed with 4% formaldehyde and stained with Alcian Blue solution to assess the proteoglycan’s synthesis by differentiated chondrocytes. After removal of the staining solution, cells were rinsed with acetic acid at 3% (v/v), neutralized with distilled water and then observed under inverted microscope.

### Preparation of dextrin-based formulations

BL granules were mixed in ODEX solution to humidify the granules and to allow the ODEX entering the granules’ pores. The final concentration of BL in HG was about 60% (w_BL_/v_HG_), since it was found to be the higher amount which does not compromise the extrusion process, the granules’ stability and the handling/moulding of the final formulation [17]. For formulations containing hMSCs, 4 × 10^4^ cells were added to ODEX solutions immediately prior crosslinking. SIS and LLKKK18 were incorporated in ADH solution before crosslinking. For the HG + BL + SIS formulations, SIS was previously sterilized by gamma irradiation (20 kGy, 2 kGy/h, at room temperature) and dissolved in sterile ADH solution, in order to obtain HGs with 4 mg/ml SIS. For HG + BL + LLKKK18 formulations, the peptide was dissolved together with ADH, then the solution was sterilized by filtration (filters 0.22 µm). The final concentration of LLKKK18 in the HG was 1 mg/ml. All formulations took about 10–15 min to gel [17].

### Surgical procedure

In this study, the bone defect model used was a critical-sized defect, which is a bone defect that does not regenerate itself [48]. All procedures were in conformity with the Directive 2010/63/EU of the European Parliament and Portuguese legislation (Portaria 1005/92), and with the approval of the Portuguese Veterinary Authorities (*Direção-Geral de Alimentação e Veterinária*). Humane endpoints were followed in accordance with the OECD Guidance Document on the Recognition, Assessment and Use of Clinical Signs as Humane Endpoints for Experimental Animals Used in Safety Evaluation [49].

**Figure 1.**
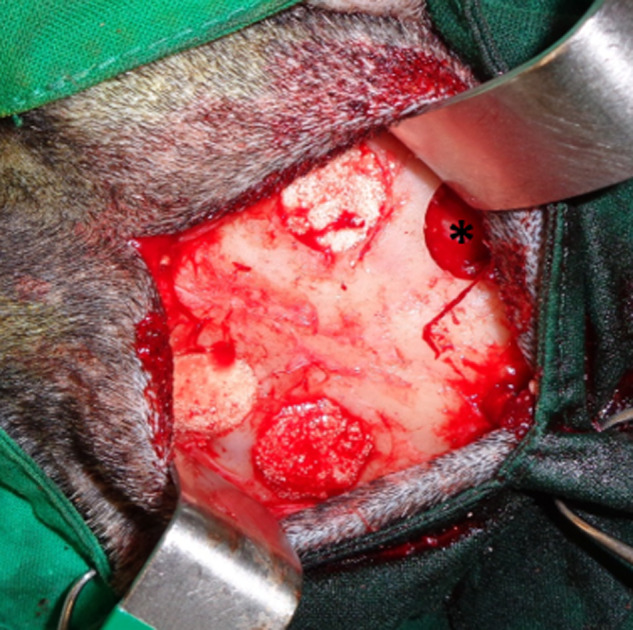
Calvarial critical-sized defects performed in the animals filled with the dextrin-based hydrogel formulations with BL granules. In each goat was performed a control defect (non-treated defect) marked with an asterisk (*)

For this experiment, 30 adult goats were used. Surgeries were conducted under general anaesthesia using intravenous injection of a combination of drugs consisting in ketamine (5 mg/kg), diazepam (0.25 mg/kg) and propofol (4 mg/kg). During the surgery, the maintenance of general anaesthesia was performed with isoflurane, after endotracheal intubation. After preparation and antisepsis of the skin with chlorhexidine 4% of the calvarial skin, an incision was made along the sagittal plan from the base of the horns until the middle of the nasal bone. The periosteum was elevated and four full-thickness calvarial critical-sized defects were made in the frontal bone with a trephine (outer ∅ 14 mm) overlying the frontal sinus, while irrigating with saline solution. For each goat, one defect was always used as control (non-treated defect) and the other ones were randomly filled with the formulations HG, BL, HG + BL, HG + BL + SIS, HG + BL + LLKKK18 and HG + BL + hMSCs (Fig. 1). The periosteum and the soft tissues were closed in layers with resorbable sutures in a continuous pattern and the skin with an intradermic suture. The goats were set free and received analgesic medication for 4 days, with flunixin meglumine and antibiotic treatment for 7 days with amoxicilin. The goats were randomly sacrificed 3, 6 and 12 weeks (*n* = 5 for each formulation at each time point) after surgery with a lethal intravenous injection of 40% sodium pentobarbital (Euthasol^®^). The frontal bones were harvested and fixed in 4% formaldehyde solution, and X-rays images were obtained. After proper fixation of the tissues, each calvarial defect was individualized using an oscillating saw for sectioning the frontal bone. Each sample was maintained in formaldehyde for further analysis.

### Micro-computed tomography analysis

Micro-computed tomography (micro-CT) scans were taken for quantitative and qualitative evaluation of new bone formed in calvarial critical-sized defects with different formulations of HG, using the μCT 100 scanner (SCANCO Medical AG, Brüttisellen, Switzerland), which operated with a cone-beam originating from a 5 μm focal-spot X-ray tube. The photons were detected by a CCD-based area detector and the projection data were computer-reconstructed into a 3072 × 3072 image matrix. A 0.5 mm aluminium filter was used for taking optimized images. For each sample, at least 1500 projections/180^°^ of X-rays (90 kVp, 200 µA, integration time 275 ms, scanning time at least 39 min) were acquired. The sample was segmented based on its grey scale values in the CT slices. The volume of interest was defined by a cylindrical contour, diameter was defined by the diameter of the drill hole (14 mm) and the height was defined by chosen the same number of slices for every sample (300 slices, 7.35 mm). The evaluation was done twice, first with a threshold of 250 to segment the BL granules only and secondly with a lower threshold of 150 to segment the new bone and the BL granules.

### Histological processing

After micro-CT analyses, samples were histologically analysed. Samples were decalcified with Surgipath decalcifier II Leica^®^, for at least 5 days, dehydrated and embedded in parafﬁn wax, in a Shandon^®^ automatic tissue processor Hypercenter XP. Consecutive 3 µm sections were cut and stained with haematoxylin and eosin. Images were acquired using a Nikon VR microscope connected to a Nikon VR digital camera DXM1200.

### Data analysis

Experimental data were presented as mean ± standard deviation. Statistical analysis of data was performed by one-way analysis of variance followed by the Bonferroni’s post-test, using the Prism^®^ version 6.1 software (GraphPad Software Inc., La Jolla, CA, USA). Significance was accepted at a *P* values <0.05.

## Results

### Multipotent MSCs characterization

**Figure 2.**
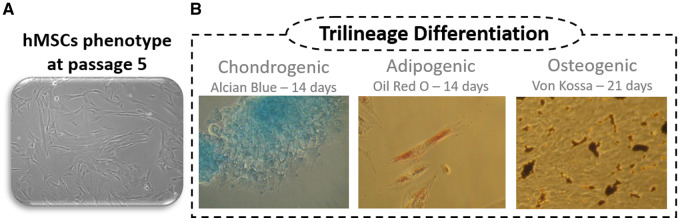
Human umbilical cord multipotent mesenchymal stromal cells (hMSCs) characterization: (**a**) hMSCs from umbilical cord at passage 5 in standard culture conditions. (**b**) Chondrogenic, adipogenic and osteogenic differentiation visualized through Alcian Blue, Oil Red O and Von Kossa histochemical staining.

hMSCs (Fig. 2a) demonstrated to be plastic adherent and to present characteristic surface markers, as assessed through flow cytometry. Results showed that 92% of the population was positive for CD90, CD105 and CD44, while ≤2% were negative for CD34, CD11b, CD19, CD45 and MHC II. Trilineage differentiation was evaluated through oil red O, Von Kossa, Alcian blue staining protocols to confirm adipogenic, osteogenic and chondrogenic differentiation, respectively. Results demonstrated successful differentiation towards the three lineages, with significant differences from undifferentiated controls (Fig. 2b).

### Surgical and post-surgical observations

The surgical procedure was fast, simple and well tolerated by the animals. The hydrogel formulations without BL granules (HG) were perfectly well-shaped to the defects. All BL containing formulations (HG + BL, HG + BL + SIS, HG + BL + LLKKK18, HG + BL + hMSCs) were easy to handle and shape into the defects, allowing the complete filling up to the periosteum level without leakage of the granules. The HG matrix facilitated the handling of the BL granules, as well as their cohesiveness and stability within the defect. Oppositely, BL only was more difficult to handle. The BL granules were mixed with blood from the animal and placed in the defect with a curette, compressing gently the granules against each other’s and against the walls of the defect to prevent their leakage of the defect. The addition of SIS and LLKKK18 did not affect the handling of the HG + BL formulations, nor the cohesiveness and stability of the BL granules within the defect.

During the post-surgery period, no surgical complications (infections, abscesses or allergic reactions) were detected, and the surgical skin incision healed without any complication. During the sample collection, skulls revealed no evidence of adverse tissue reaction or infection.

### Performance of the dextrin-based HG in bone regeneration of calvarial critical-sized defects

#### Micro-CT analysis

**Figure 3.**
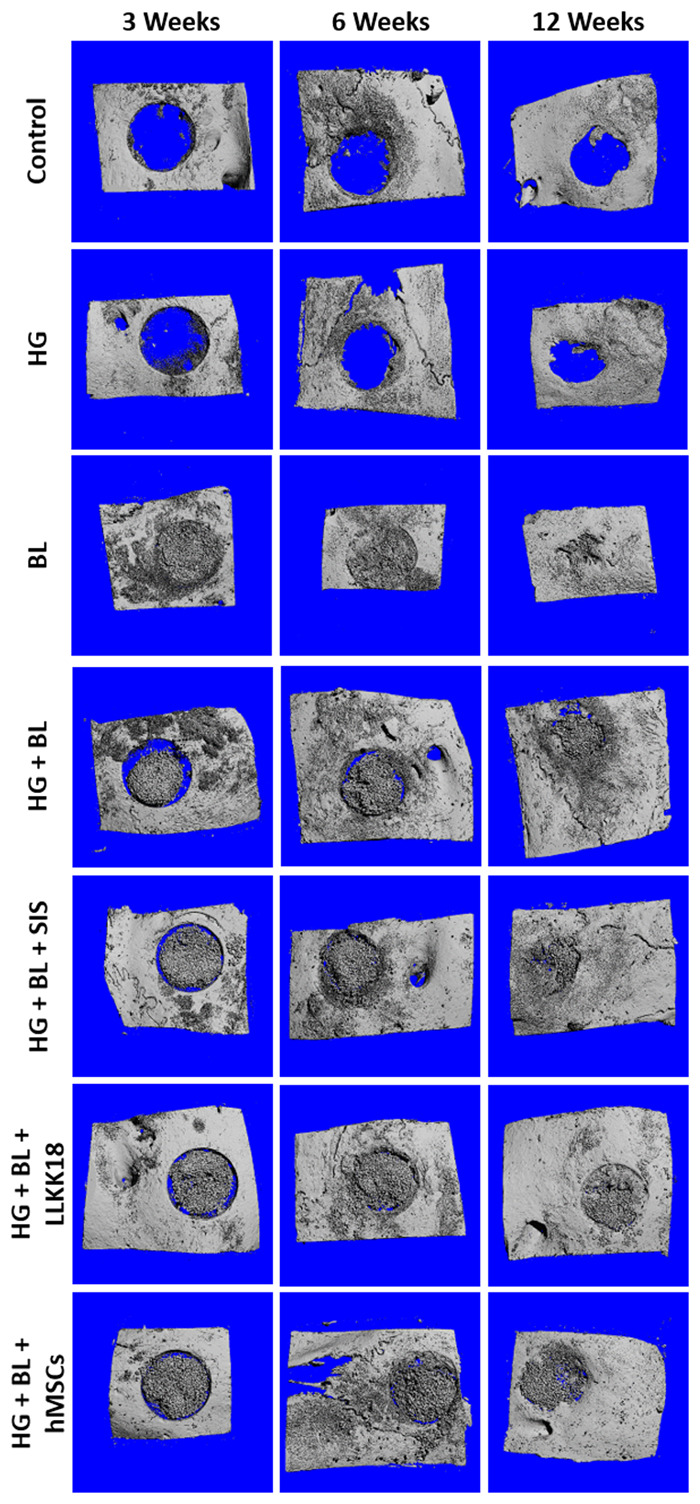
Representative micro-CT reconstruction images of calvarial critical-sized defects, after 3, 6 and 12 weeks, for the different dextrin-based hydrogel (HG) formulations.

Micro-CT reconstruction was carried out for the calvarial bone samples at each time point to provide a comprehensive visualization of the whole bone region where the defect was created, as shown in Fig. 3. The images allowed to verify that non-treated defects (control) did not heal over time, only a small amount of bone formation being noted in the periphery of the defect. The same was observed for the HG-treated defects. The addition of osteoconductive BL granules promoted new bone formation over time; at 12 weeks post-surgery, it is no longer possible to identify the initial defect, with complete osseointegration being observed. In defects treated with HG + BL formulation, an empty space in peripherical areas of the defects was observed, a progressive osteointegration of the formulation occurring over time, although slower than in defects treated with BL granules only. These results suggest that HG delayed the onset of the bone tissue ingrowth process, not preventing, however, the formation of new bone, neither affecting the BL granules’ osteoconductivity.

**Figure 4.**
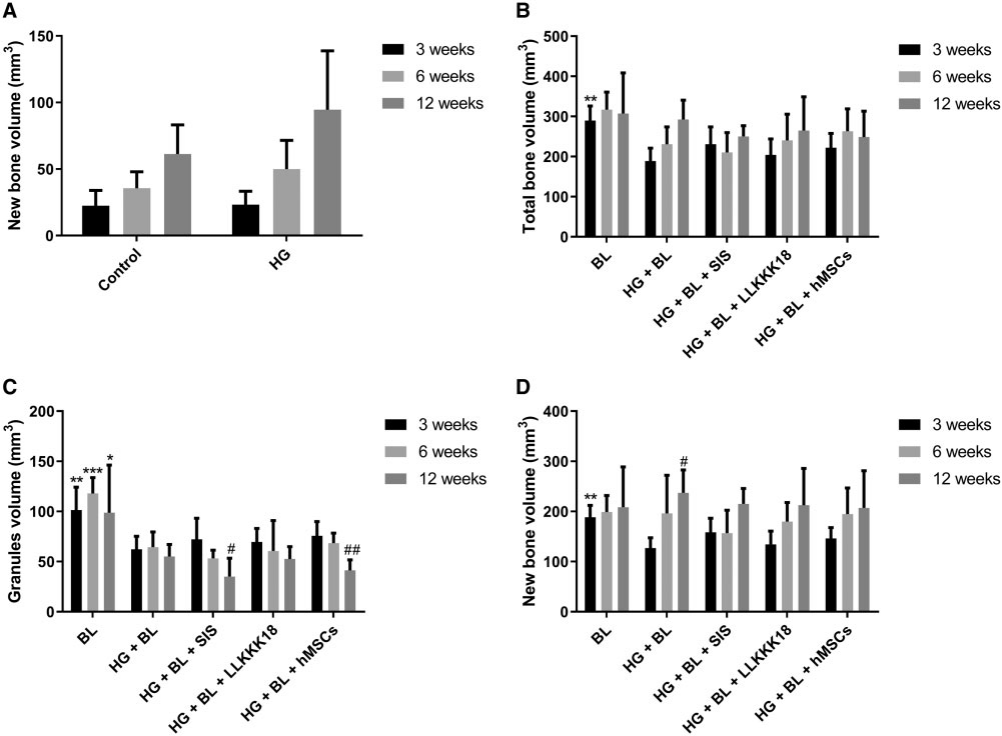
Results of micro-CT analysis for critical-sized calvarial defects, after 3, 6 and 12 weeks: (**a**) quantification of the new bone volume formed in the defects treated with the dextrin-based hydrogel (HG) alone and the non-treated defect (control). (**b**) Quantification of the total bone volume (new bone and granules volume), (**c**) the granules volume and (**d**) the new bone volume in the defects treated with BL granules alone or incorporated into HG matrix (HG + BL) and its combination with 4 mg/ml of SIS (HG + BL + SIS), 1 mg/ml of LLKKK18 (HG + BL + LLKKK18) or hMSCs (HG + BL + hMSCs). Data are presented as mean ± SD (*n *=* *5 replicates per group) and were analysed by one-way ANOVA followed by Bonferroni’s *post hoc* test: *P <* *0.05, **P <* *0.01 and ***P <* *0.001 vs. HG + BL treatment, ^#^*P *<* *0.05 and ^##^*P *<* *0.01 vs. 3 weeks (intra group).

The micro-CT bone quantification (Fig. 4a) confirmed that in the non-treated defects and in those treated with HG only, little amount of bone was formed, being the bone volume similar over time in both defects. In relation to BL and HG + BL-treated defects, the granules volume and the total bone volume were quantified, allowing the calculation of the new bone volume. At 3 weeks, the mean value of total bone volume in the HG + BL group was 35% lower (*P *<* *0.01) than observed in the BL group, converging overtime to similar average values (Fig. 4b). For the BL-treated defects, the total bone volume was almost constant over time. Relating to the volume of BL granules, the same amount of BL granules was applied in the defects treated with BL only (with autologous blood) and with HG + BL. The results shown that the amount of BL was practically the same over time (Fig. 4c) in each group, BL and HG + BL. However, the micro-CT quantification revealed that the volume of granules in defects treated with HG + BL was significantly lower (42.8% ± 3.6) at all time points (*P *<* *0.05) in comparison with BL group. Considering the new bone volume (Fig. 4d), it was similar over time in the BL-treated defects. In the case of the HG + BL-treated ones, the new bone volume was lower (about 37%) at 3 weeks when compared with the BL group (*P *<* *0.01). However, a 14% increase in new bone deposition was noticed for HG + BL at 12 weeks in comparison with BL-treated defects.

#### Histological analysis

**Figure 5.**
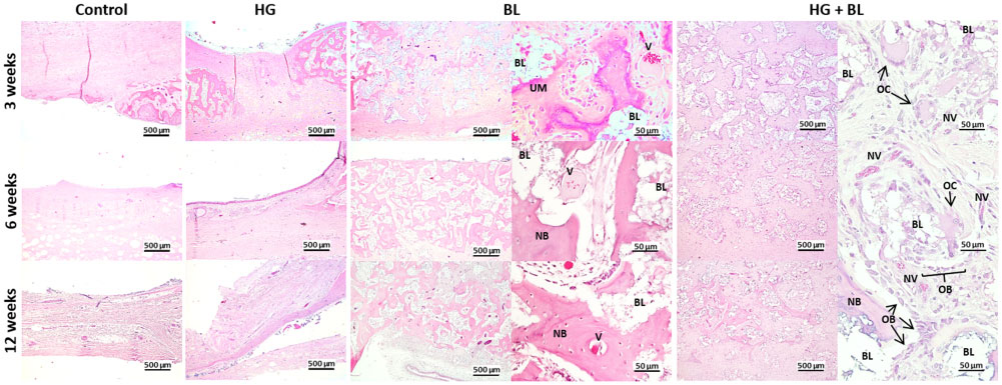
Haematoxylin- and eosin-stained histological sections from calvarial critical-sized defects of decalcified samples. BL, Bonelike^®^ granules; NB, new bone; NV, new vessels; V, vessels; OB, osteoblasts; OC, osteoclasts; UM, unmineralized bone.

A complementary histological analysis of the decalcified tissue sections stained with haematoxylin and eosin (Fig. 5) was conducted at each time point. The non-treated defects (control) were mainly occupied by dense fibrous tissue with residual bone formation at the margins of the defect, at all tested times. From 6 weeks post-surgery, remodelled bone was also found in the edges of the defect. For the defects treated with the HG, at 3 weeks no traces of HG were observed within the defects, and no signs of inflammatory response, nor bone necrosis or fatty infiltrate were observed. The defects were mainly occupied by dense fibrous tissue with residual bone formation at the margins of the defect, in all tested times, as observed in non-treated defects.

The defects treated only with BL granules revealed a growing increase of newly formed bone in the entire defect between the granules, over time, accompanied by a decrease in fibrous tissue. At 3 weeks, osteoid formation in the intergranular space was found, and in some spots mineralized matrix was also observed. Intragranular spaces were colonized by fibrous tissue. At 6 and 12 weeks, the new bone matrix was mineralized almost in the entire defect.

The defects treated with HG + BL presented a delay in the deposition and formation of new bone, in comparison to BL-treated counterpart. At 3 weeks, the intergranular space was composed mostly by fibrous tissue. Osteogenic cells around and/or inside the granules were also observed, as well as new vessels. At 6 weeks, the entire defect is still composed mostly by fibrous tissue. At 12 weeks, some spots of bone matrix were observed, most of them composed by unmineralized matrix and several new vessels could be observed, close to the BL granules.

### Performance of the formulations complemented with SIS, LLKKK18 and hMSCs

#### Micro-CT analysis

Micro-CT reconstruction was carried out for the calvarial bone samples at each time point as represented in Fig. 3. The images allowed to verify that the defects treated with HG + BL + SIS, HG + BL + LLKKK18 and HG + BL + hMSCs display an empty space in peripherical areas of the defects, which decreased over time, concomitant with a progressive osseointegration of the formulation (Fig. 3), as observed in HG + BL-treated defects.

The micro-CT quantification results (Fig. 4) demonstrated that the new bone and granules volumes obtained for HG + BL + (SIS, LLKKK18 or hMSCs) was not significantly different from those observed with HG + BL, for each time point. These results suggest that SIS, LLKKK18 and hMSCs did not significantly enhance the new bone formation.

#### Histological analysis

**Figure 6.**
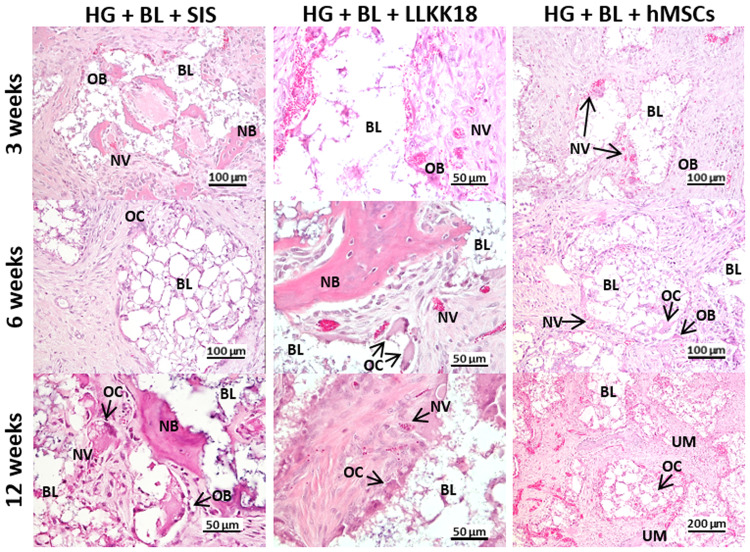
Haematoxylin- and eosin-stained histological sections of decalcified samples from calvarial critical-sized defects treated with 4 mg/ml of SIS (HG + BL + SIS), 1 mg/ml of LLKKK18 (HG + BL + LLKKK18) or hMSCs (HG + BL + hMSCs). BL, Bonelike^®^ granules; NB, new bone; NV, new vessels; OB, osteoblasts; OC, osteoclasts; UM, unmineralized bone.

Histological analysis of decalcified tissue sections was conducted at each time point (Fig. 6). The results shown that the incorporation of SIS, LLKKK18 and hMSCs in HG + BL formulations seemed to not accelerate the new bone formation, under the tested conditions, since histological observations were similar those observed in HG + BL samples (Fig. 5).

## Discussion

Despite the high innate regenerative capacity of bone, critical-sized defects fail to heal and remain a clinical challenge, both in orthopaedics and maxillo-facial fields. Healing of such defects needs the formation of large amounts of bone in an environment often rendered hostile to osteogenesis by damage of the surrounding soft tissues and vasculature [50]. In this work, full-thickness 14 mm Ø frontal bone osteotomies were created in goats, to obtain critical-sized defects.

BL is a SBS (Biosckin S.A.) constituted of modified calcium phosphate and controlled proportions of HAP, tricalcium phosphate (TCP) and ionic species, mimicking the structural and chemical composition of the human bone [4, 51]. Several studies on orthopaedics, maxillofacial and dental applications, performed in humans and animals, have reported the BL osteoconductive properties [6, 51–55]. Moreover, such studies have shown that BL is rapidly osteointegrated after implantation and displays a sustained controlled resorption, while new bone formation occurs. The results obtained in this study confirmed the remarkable potential of osteoconductivity and osseointegration of BL, since the granules and empty spaces were quickly colonized and osteointegrated in bone tissue, being able to regenerate critical-sized defects. At 3 weeks, the empty inter- and intra-granular spaces were filled with new bone, the formation of additional new bone being henceforward dependent of granules’ resorption. The use of this material in the clinical setting requires autologous blood as a carrier for the granules, that is not satisfactory. A more adequate and less invasive vehicle to improve the per-operative handling properties, stabilizing the granules in large defects or unstable sites, and avoiding the migration of particles from the site of implantation is required. For that, we proposed the use of the HG as a carrier and stabilizer of BL granules [15].

In this study, it was shown that the HG, alone, did not stimulate the bone healing process of critical-sized defects. Also, it was also not harmful to the native bone, since no inflammatory reaction or necrotic tissue were observed. Furthermore, the combination of HG and BL granules improved the handlings properties of the granules, as wells as its application in the defects. Concerning to the bone healing process, such combination resulted in a short delay in bone tissue ingrowth. This delay can be explained by the considerable decrease of BL granules volume observed in HG + BL formulations compared with treatment with BL alone. Prior to implantation, the BL granules were mixed with ODEX solution as to wetting the granules and to allow the ODEX permeating into the granules’ pores. The pH of this solution is about 3.0 and after BL granules addition, the pH increases to 4.7. When ADH solution is added, the pH increases to 5.2 and remains around this value until complete gelation of the formulation. This acidic environment resulted in an acceleration of the initial resorption of the BL minerals, mainly of TCP phase, which is more soluble than HAP [3]. Other studies reported the enhanced degradation of calcium phosphate-derived granules induced by polymeric solutions/hydrogels [56–58]. Daculsi *et al.* [59] studied specifically the biphasic calcium phosphate granules’ surface interactions with hydroxypropyl methyl cellulose (HPMC) by high-resolution transmission electron microscopy. In this study, it was observed hydrolysis from the surface to about 13 nm into the HAP crystals and occasional dissolution with precipitation of β-TCP crystals. The HAP surface and/or the β-TCP surface remained unchanged after a short incubation with HPMC (1 week) up to 1 year. Further experiments are needed to better assess the BL surface interactions with the HG, mainly with ODEX component, at the crystal level. Considering that the BL supports the bone healing by osteoconduction, a decreasing in granules volume in earlier times resulted in a reduction of new bone deposition at 3 weeks. However, this effect was mitigated at 6 and 12 weeks. Thus, the HG did not impair the formation of new bone over time, neither affected the BL granules’ osteoconductivity.

The creation of bone defects and subsequent filling of the defect triggers an inflammatory response, leading to a decrease of pH, at the implanted site. However, the acidic solution of ODEX can aggravate the pH of the lesion, delaying the inflammation-regeneration transition, that can also explain the delayed bone formation observed at 3 weeks.

When the BL granules were implanted into the bone defect, inter- and intragranular spaces were immediately colonized by cells. However, when BL was combined with HG, the matrix had to be first degraded from granules’ surface for cells to be able to adhere to osteoconductive granules, proliferate, differentiate and deposit new bone. This could also contribute for the short delay in bone tissue ingrowth observed in the defects treated with HG + BL at 3 weeks, when compared with the defects treated only with BL. A delay in bone tissue ingrowth have also been described with fibrin glue [60] and HPMC-Si hydrogel [56, 61] with biphasic calcium phosphate granules. Additionally, in the HG + BL formulations, the HG moved towards the periphery of the defects in the earlier times, creating a gap between the defect wall and the BL granules. Once the HG was reabsorbed, bone regeneration proceeded normally over time, simultaneously with the osseointegration process.

Besides allowing to obtain mouldable and injectable bone substitutes and granules’ cohesiveness, hydrogels can also modulate cell colonization, as well as the bone healing process, since these systems allow the incorporation of several bioactive agents able to improve cell adhesion, osteoinductivity and osteointegration of the IBSs [9, 10]. Different strategies have been reported to enhance the bioactivity of the IBSs-based hydrogels. Some authors have added ECM components into the hydrogel matrix, such as collagen [62, 63], hyaluronic acid [64], immobilized covalently RGD peptides on hydrogel [65] or even used peptide- and hyaluronic acid-based hydrogels [57, 66] to improve cell adhesion and hence to modulate cell events (cell proliferation, differentiation and migration). Others have included bone morphogenetic protein-2 [66–70] or dexamethasone [70, 71] to obtain osteoinductive IBSs. IBSs with antimicrobial features have also been reported [64, 71, 72]. In this study, several approaches were tested to improve the performance of the HG + BL formulations on the regeneration of critical-sized bone defects. Such approaches included the incorporation of ECM-based signals (degradation products from ECM-SIS), angiogenic factors (LLKKK18) and cell therapy (hMSCs). However, no significant differences in new bone formation process where observed in comparison to HG + BL group. It is possible that the acid environment promoted by the HG, as discussed above, has nullified the performance of these bioactive agents. Moreover, since LLKKK18 and SIS where just mixed in the relatively porous HG network, we hypothesize that a quick burst release may have occurred, such that a sustained and prolonged effect was not observed. HG-based systems with a controlled release of the bioactive agents should be tested and their *in vivo* responses further assessed in relation to non-immobilized systems ones. Furthermore, the concentration of the bioactive agents used may be relevant. The LLKKK18 concentration was based on a previous work [34], where 1 mg/ml LLKKK37 in collagen sponges was used, resulting in an enhanced bone regeneration in the critical-sized calvarial defect, using a rat model. In spite of the present study intended to explore the effect of LLKKK18 on the bioactivity of HG + BL formulations, its molecular mechanism on bone regeneration process needs to be studied, since no data were found. Regarding SIS, the concentration used (4 mg/ml) resulted from the maximum concentration that it was possible to solubilize in ADH solutions.

## Conclusion

In conclusion, the HG allowed to improve the handling properties of granular ceramics. It delayed the onset of bone tissue ingrowth process, but did not affect BL granules’ osteoconductive properties neither impaired the bone repair/regeneration process of critical-sized defects, demonstrating the HG’s ability to act as a biocompatible mouldable carrier. The HG also allowed the incorporation and delivery of diverse bioactive agents, including short half-life agents. However, the association of the tested agents did not improve the bone regeneration process over 12 weeks. Human clinical trials will be performed to validate the potentiality of the HG as a carrier of bone graft granules under clinical procedures.

## Funding

This work was funded by the project ‘DEXGELERATION – Advanced solutions for bone regeneration based on dextrin hydrogels’ (Norte-07-0202-FEDER-038853). It was also funded by FCT under the scope of the strategic funding of UID/BIO/04469/2013 and UID/BIM/04293/2013 units and COMPETE 2020 (POCI-01-0145-FEDER-006684), BioTecNorte operation (NORTE-01-0145-FEDER-000004) and NORTE-01-0145-FEDER-000012 funded by FEDER under the scope of Norte2020 - Programa Operacional Regional do Norte. Isabel Pereira and Ana Rita Caseiro were supported by the grants FRH/BD90066/2012 and SFRH/BD/101174/2014, respectively, from FCT, Portugal.

*Conflict of interest statement*. None declared.
